# A visual mining analysis of middle meningeal embolization and other factors associated with recurrence requiring re-operation in subdural hematomas: a single-center series

**DOI:** 10.1007/s00701-025-06737-8

**Published:** 2025-12-20

**Authors:** Marco Battistelli, Marika Vezzoli, Iacopo Valente, Massimo Benenati, Giuseppe Garignano, Andrea Alexandre, Ludovico Agostini, Samuele Santi, Ottavia Giovinazzo, Leonardo Nardini, Federico Costa, Giorgio Quintino D’Alessandris, Manuela D’Ercole, Alessandro Izzo, Alessandro Rapisarda, Francesco Signorelli, Nicola Montano, Simona Gaudino, Alessandro Olivi, Alessandro Pedicelli, Filippo Maria Polli, Francesco Doglietto

**Affiliations:** 1 Neurosurgery, Università Cattolica del Sacro Cuore, Rome, Italy; 2 Neurosurgery, Fondazione Policlinico Universitario Agostino Gemelli IRCCS, Largo A. Gemelli, 8, 00168 Rome, Italy; 3 Department of Molecular and Translational Medicine, University of Brescia, Brescia, Italy; 4 UOSA Neuroradiologia Interventistica, Fondazione Policlinico Universitario A. Gemelli IRCCS Roma, Roma, Italy; 5 Dipartimento Di Diagnostica Per Immagini, Radioterapia, Oncologia Ed Ematologia, Fondazione Policlinico Universitario A. Gemelli IRCCS, Rome, Italy; 6 Università Cattolica del Sacro Cuore, Rome, Italy

**Keywords:** Case-series, Chronic subdural hematoma, Middle meningeal artery embolization, Markwalder grading scale, Platelet count, Recurrence requiring reoperation

## Abstract

**Purpose:**

to comprehensively and hierarchically assess risk factors for recurrence requiring reoperation (RrR) in chronic subdural hematoma (cSDH) in the era of middle meningeal artery embolization (MMAE).

**Methods:**

Patients treated for a cSDH from January 2019 to October 2024 at Fondazione Gemelli research hospital were considered for inclusion. Clinical, coagulation, radiological, and treatment factors were recorded. MMAE was performed systematically from October 2022, using polyvinyl alcohol (PVA) particles injected directly from the main trunk of MMA.

The dataset comprised 45 quantitative and qualitative variables for each cSDH. Variables showing statistical significance (p-value < 0.05) were selected as covariates in two supervised learning frameworks to predict the RrR (outcome, *Y*): (*i*) Classification and Regression Tree (CART) and (*ii*) Random Forest (RF) classifier.

**Results:**

500 patients were eligible and 233 were included, resulting in 283 treated cSDHs (mean follow-up: 119 days); 129 underwent adjuvant MMAE. 50 cSDH had a RrR (mean time to recurrence: 47 days), of which 41 (82%) in the non-embolized group and 9 (18%) in the embolized group (p-value < 0.001). Adjuvant embolization was the strongest factor associated with RrR, significantly reducing the risk for reintervention. Markwalder grading scale, preoperative cSDH volume, and platelet count (PLT) are strong predictors in non-embolized patients. A critical PLT cut-off of 229 × 10^9^/L strongly impacts RrR risk for substantial cSDH volumes.

**Conclusions:**

The present results support the routine use of MMAE and the correction of PLT in relation to cSDH volume.

## Introduction

Chronic subdural hematoma (cSDH) is one of the most prevalent neurosurgical diseases, with an estimated frequency ranging from 1 to 14 cases per 100,000 individuals [8, 16]. It predominantly afflicts individuals over the age of 65 [8, 11, 16, 23], with an estimated incidence of 80 cases per 100,000 elderly people [8]. Given the aging population, it is anticipated that by 2030, it will become the most prevalent cranial neurosurgical condition among adults [5, 8]. Surgical evacuation has proven to be limited by a concerning recurrence rate, which reaches up to 50% of cases, regardless of the surgical technique [5, 8, 11, 16, 23, 29].

Several factors are associated with a higher risk of recurrence. Nakaguchi et al. first introduced a classification of the internal architecture and density of cSDH based on the risk of recurrence, with the separated type representing the highest risk and the laminar type the lowest [22]. More recently, Stanišic et al. integrated the Nakaguchi classification with volumetric parameters, highlighting that a preoperative volume (V1) greater than 130 mL and a postoperative residual volume (V2) greater than 80 mL are associated with an increased risk of relapse in cSDH evacuated by the burr hole technique [29]. Additionally, postoperative pneumocephalus, baseline brain atrophy, and bilateral haematomas have been documented as predictive of cSDH recurrence [15, 24]. The patient's coagulation profile is another critical factor linked to the risk of cSDH recurrence. Eagle et al. constructed a chi-squared automatic interaction detection model in which preoperative platelet count (PLT) emerged as the most significant factor influencing the risk of cSDH relapse in patients treated with surgical evacuation, with a critical cut-off estimated at 157 × 10^9^/L [10]. Furthermore, antiplatelet therapy is a well-established factor influencing the risk of recurrence [10, 33], whereas the evidence regarding the role of aspirin is conflicting [10, 13].

Recently, MMA embolization (MMAE) has emerged as a promising therapeutic option [19], and its use is now well established, both as a stand-alone procedure [14, 17] and as an adjunct to standard surgical evacuation, but remains debated [5, 8, 11, 23].

Despite numerous studies in the existing literature that document various risk factors for the recurrence of cSDH, there is a lack of recent studies that comprehensively consider them. This study was designed to investigate the role of various factors associated with the risk of cSDH recurrence requiring reoperation (RrR), including MMAE, which has recently become the standard of care at our center, in adjuvant to surgical evacuation and drain.

## Methods and materials

### Study population

Patients admitted to the University Hospital Policlinico Agostino Gemelli in Rome for the treatment of cSDH between January 2019 and October 2024 were considered for inclusion. The Institutional data warehouse office identified patients' digital charts utilizing the ICD codes 432.1 and 852.2. Forty-five records were excluded as they pertained to patients treated for aSDH, hygroma, or empyema. Beginning from October 2022, MMAE was consistently proposed as an adjunctive treatment to standard surgical evacuation. The group was compared to a historical cohort of patients from January 2019 who received standard surgical evacuation. A single-center, retrospective analysis of the records was conducted.

Surgical treatment did not change between 2019 and 2024 and included both burr hole and minicraniotomy, with a subdural drain usually positioned in both cases and removed the day after surgery. A postoperative CT scan was obtained within 24 h of surgery. Patients were excluded if they had no pre-operative CT (diagnosis with MRI only), presented with secondary cSDH, were treated with MMAE only, or had no follow-up data. The records were extracted from the Progressive Advancement in Neurosurgery Data and Analysis Database (PANDA) registry, which is registered on ClinicalTrials.gov (ID: NCT06835634). The case series has been reported in accordance with PROCESS 2023 guidelines [20]. All participants gave their informed consent for the collection and use of their data for publication, and the study was carried out in accordance with the Declaration of Helsinki.

### Embolization technique

MMAE was performed under local anaesthesia or, in non-cooperative patients, under conscious sedation. Following vascular access, the MMA was selectively catheterized using a 0.021-inch inner diameter microcatheter. Embolization was carried out by injecting 150–250 micron polyvinyl alcohol (PVA) particles (Contour TM Boston Scientific) directly into the main trunk of the MMA, proximal to the foramen spinosum, to take advantage of the higher blood flow and facilitate distal particle distribution. Embolization was performed on each side affected by the pathology. To minimize potential era-bias, provider variability and training effect biases, four experienced neurointerventional radiologists (I.V., G.G., A.A., A.P.) conducted all procedures while maintaining a consistent technique throughout the study duration.

### Outcome

The primary outcome was the recurrence rate, defined as any reaccumulation of hematoma associated with lateralizing neurologic signs and/or symptoms and/or cognitive dysfunction. Therefore, the reoperation rate was a real reflection of the underlying pathology and was defined as RrR. The included cSDHs were stratified into the RrR group and the non-RrR group.

### Data collection

The data were recorded at four distinct time-points: admission, treatment, discharge, and final follow-up. The following data were collected at admission: age, gender, BMI, trauma and/or seizure history, antiplatelet (AP) and/or anticoagulation (AC) therapy (stratified as NAO/DOAK, AVK/TAO, P2Y12, ASA) [33], double AP and/or AC therapy, Markwalder grading scale (MGS) [25], modified Rankin Scale (mRS), Activated Partial Thromboplastin Clotting Time (aPTT), INR, PLT [10], midline shift, maximum cSDH thickness, brain atrophy [24], Nakaguchi grade [22], V1 and extended Nakaguchi classification grade [29]. The collected treatment parameters included: surgery side, surgery bilaterality [15], surgery technique [9], surgery during AC and/or AP wash-out, drain use [33], embolization, and embolization during AC and/or AP wash-out [13]. The following discharge parameters were recorded: length of hospital stay (LOS), MGS, mRS, V2, and pneumocephalus volume > 20% of V2 [24]. The collected parameters at final follow-up were: MGS, mRS, complications other than recurrence, timing of re-assumption of AC and/or AP therapy, last follow-up hematoma volume (V3), residue thickness and RrR rate. Follow-up length was defined in relation to the date of recurrence in the RrR group and the date of the last CT scan in the non-RrR group.

Volumes were measured on a non-contrast brain CT scan (GE LightSpeed Pro 64, GE Medical Systems, Milwaukee, USA), axial slices with a thickness of 2.5 mm. The data were processed using a workstation (Advanced Workstation; GE Medical Systems, Milwaukee, WI) and analysed by a neuroradiologist with 10 years of experience, using software that enabled manual outlining of subdural hematomas.

### Data preprocessing, statistical analysis, and the machine learning framework

The dataset comprised 45 quantitative and qualitative variables, including both nominal and ordinal types, corresponding to 283 subdural hematomas, which served as the statistical units of this study.

Descriptive statistics were computed overall and stratified by the clinical outcome. For continuous variables, data were summarized using the mean ± standard deviation (SD), median with the first (Q1) and third quartiles (Q3), and range (minimum to maximum). Categorical variables were described through absolute and relative frequencies. For all variables, the number of missing observations (N-Miss) and non-pertinent entries (NP) were reported; these were systematically excluded through predefined filtering procedures to mitigate potential biases and uphold the assumptions underlying inferential testing.

Group comparisons between subdural hematomas with and without recurrence were conducted using non-parametric Kruskal–Wallis rank-sum tests for continuous variables, Fisher’s exact tests for nominal variables, and Cochran–Armitage trend tests for ordinal predictors. Variables showing statistical significance (*p*-value < 0.05) were selected as covariates in two supervised learning frameworks: (*i*) Classification and Regression Tree (CART) [1, 3, 7, 32] and (*ii*) Random Forest (RF) [2] classifier: The aim was to identify the most influential predictors, characterizing the key determinants of outcome (recurrence requiring reoperation).

The CART model was estimated using a classification approach on clinically relevant predictors, with a maximum tree depth set to 8 and a complexity parameter fixed at 0.01 to control overfitting. The resulting tree was visualized, showing node-wise prediction probabilities and indicators of classification purity.

To enhance predictive robustness and account for potential instability in single-tree models, a RF was also implemented. By aggregating the predictions of thousands of decorrelated decision trees trained on bootstrap samples and random subsets of predictors, RF mitigates overfitting and captures complex, non-linear interactions between variables. This ensemble approach provides a more stable and generalizable estimate of feature importance and recurrence risk [6]. RF implemented in R does not support missing values within predictor variables during model training. This limitation arises because the internal mechanics of decision tree algorithms rely on recursive binary splits over complete observations, and incomplete data would prevent the proper evaluation of the Gini impurity measure that drives node partitioning [30]. To address this, missing data were imputed using multivariate imputation by chained equations (MICE) [4]. Using clinically relevant predictors, imputation was performed across five datasets in five iterations, utilizing either stochastic regression or classification trees, depending on the variable type. This approach preserves statistical power by avoiding case-wise deletion and mitigates potential bias introduced by non-random patterns of missingness, assuming data are missing at random (MAR) [26]. The Random Forest classifier was trained on the imputed dataset with 10,000 trees. For each tree, bootstrap sampling was applied to the observations, and at each node, a random subset of predictors equals to $\sqrt{\#predictors}$ was considered for splitting, thereby introducing stochasticity and reducing overfitting. To clarify the internal decision-making process of the RF, graphical representations of variable importance and partial dependence plots (PDPs) were obtained to provide insights into the model learned structure and the role of individual predictors. Variable importance was computed based on the mean decrease in Gini impurity and normalized to the highest-ranked variable to yield a relative importance score (0 less important, 100% most important). These results were visualized using a lollipop graph, where the *x*-axis reports the relative importance (%) of each variable, and the *y*-axis lists the clinical variables included in the model, ranked in descending order of importance from top to bottom.

Additionally, to interpret the functional relationship between the three most important variables and the predicted probability of recurrence, PDPs were constructed. Two-dimensional contour plots (based on pairs of variables, in static and interactive format) and three-dimensional surface plots (based on triplets of variables) were generated using the most influential predictors to enhance the interpretability of their joint effects on the outcome [21].

Discriminative ability of the two methods was quantified using the area under the receiver operating characteristic (ROC) curve (AUC). An optimal cut-off was identified using Youden’s J statistic, and classification performance was summarized via accuracy.

All analyses were conducted in R (v 4.4.1; R Foundation for Statistical Computing, Vienna, Austria).

## Results

### Study population

**Fig. 1:**
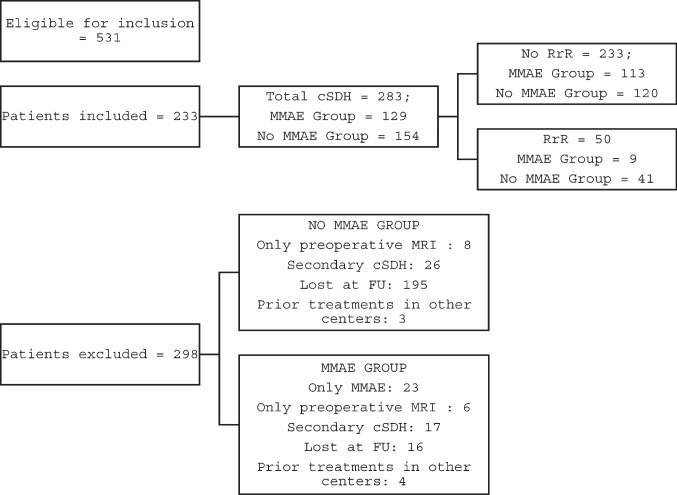
Flow-chart of the inclusion process. RrR: recurrence requiring reoperation; MMAE: middle meningeal artery embolization; aSDH: acute subdural haematoma; FU: follow-up

The inclusion process is shown in Fig. 1. Of 531 screened patients, 233 were included. In 50 cases, cSDH was bilateral. Thus, a final sample of 283 cSDH was considered. 129 (45.6%) cSDH underwent adjuvant MMAE and 154 (54.4%) were treated surgically only, with burr-holes or mini-craniotomy technique. Fifty cSDH had a RrR, of which nine (18%) were in the embolized and forty-one in the non-embolized cSDH (82%) (*p*-value < 0.001). Mean time to recurrence was 46.9 (SD 78.25) days, while the mean follow-up length in the non-RrR group was 130 (SD 118.8) days. The following factors differed significantly between RrR and non-RrR groups (Table 1 of Supplementary Materials): gender (*p*-value = 0.001), MGS at admission (*p*-value = 0.010), mRS at admission (*p*-value = 0.028), PLT at admission (*p*-value = 0.026), preoperative cSDH volume (*p*-value = 0.002), points according to Oslo Grading System (*p*-value = 0.037), surgery on AP and/or AC (*p*-value = 0.016), embolization (*p*-value < 0.001), V2 (*p*-value = 0.026).

**Table 1:** Descriptive analysis

	No RrR (*N* = 233)		RrR (*N* = 50)		Total (*N* = 283)		*p*-value
Clinical-Biochemical Parameters at Admission							
Age							0.062
Mean (SD)	76.20 (10.53)		79.10 (8.46)		76.71 (10.24)		
Median (Ǫ1, Ǫ3)	78.00 (72.00,		80.50 (75.00,		79.00 (72.00,		
	83.00)		83.75)		83.00)		
Range	33.00—97.00		57.00—93.00		33.00—97.00		
Gender							***0.001***
Female	69 (29.6%)		4 (8.0%)		73 (25.8%)		
Male	164 (70.4%)		46 (92.0%)		210 (74.2%)		
BMI							0.146
N-Miss	30		4		34		
Mean (SD)	25.90 (3.53)		25.02 (2.44)		25.74 (3.37)		
Median (Ǫ1, Ǫ3)	25.26 (23.58,		24.22 (23.46,		25.14 (23.53,		
	27.76)		26.12)		27.44)		
Range	16.82—36.73		19.53—30.48		16.82—36.73		
Trauma history							0.077
No	83 (35.6%)		25 (50.0%)		108 (38.2%)		
Yes	150 (64.4%)		25 (50.0%)		175 (61.8%)		
Seizures history							1.000
No	216 (92.7%)		47 (94.0%)		263 (92.9%)		
Yes	17 (7.3%)		3 (6.0%)		20 (7.1%)		
Markwalder grading scale							***0.010***
0	1 (0.4%)		0 (0.0%)		1 (0.4%)		
1	44 (18.9%)		6 (12.0%)		50 (17.7%)		
2	171 (73.4%)		34 (68.0%)		205 (72.4%)		
3	14 (6.0%)		8 (16.0%)		22 (7.8%)		
4	3 (1.3%)		2 (4.0%)		5 (1.8%)		
mRS							***0.028***
0	1 (0.4%)		0 (0.0%)		1 (0.4%)		
1	6 (2.6%)		2 (4.0%)		8 (2.8%)		
2	67 (28.8%)		9 (18.0%)		76 (26.9%)		
3	93 (39.9%)		18 (36.0%)		111 (39.2%)		
4	47 (20.2%)		11 (22.0%)		58 (20.5%)		
5	19 (8.2%)		10 (20.0%)		29 (10.2%)		
First anti-platelet and/or anti-coagulation therapy							0.767
0	114 (48.9%)		27 (54.0%)		141 (49.8%)		
1	25 (10.7%)		3 (6.0%)		28 (9.9%)		
2	17 (7.3%)		2 (4.0%)		19 (6.7%)		
3	11 (4.7%)		3 (6.0%)		14 (4.9%)		
4	66 (28.3%)		15 (30.0%)		81 (28.6%)		
Second anti-platelet and/or anti-coagulation therapy							0.727
2	3 (1.3%)		1 (2.0%)		4 (1.4%)		
3	3 (1.3%)		0 (0.0%)		3 (1.1%)		
4	3 (1.3%)		2 (4.0%)		5 (1.8%)		
NP	224 (96.1%)		47 (94.0%)		271 (95.8%)		
Double anti-platelet and/or anti-coagulation therapy							0.450
No	224 (96.1%)		47 (94.0%)		271 (95.8%)		
Yes	9 (3.9%)		3 (6.0%)		12 (4.2%)		
INR							0.472
N-Miss	16		0		16		
Mean (SD)	1.08 (0.16)		1.09 (0.13)		1.09 (0.16)		
Median (Ǫ1, Ǫ3)	1.05 (1.01,		1.06 (1.00,		1.05 (1.01,		
	1.10)		1.12)		1.11)		
Range	0.90—2.10		0.93—1.60		0.90—2.10		
aPTT							0.488
N-Miss	16		0		16		
Mean (SD)	29.78 (6.38)		30.07 (5.79)		29.84 (6.26)		
Median (Ǫ1, Ǫ3)	29.50 (25.30,		29.00 (25.92,		29.20 (25.40,		
	33.00)		33.90)		33.45)		
Range	20.70—64.70		20.40—44.70		20.40—64.70		
PLT							***0.02***
N-Miss	10		0		10		
Mean (SD)	224.88 (79.74)		195.06 (55.27)		219.42 (76.64)		
Median (Ǫ1, Ǫ3)	222.00		211.50		216.00		
	(176.00,		(160.50,		(172.00,		
	259.00)		221.75)		252.00)		
Range	59.00—633.00		32.00—305.00		32.00—633.00		
*Radiographic Parameters at Admission*							
cSDH Bilaterality							1.000
Bilateral	82 (35.2%)		18 (36.0%)		100 (35.3%)		
Monolateral	151 (64.8%)		32 (64.0%)		183 (64.7%)		
Midline shift (mm)							0.101
Mean (SD)	7.54 (4.70)		8.88 (5.22)		7.78 (4.82)		
Median (Ǫ1, Ǫ3)	7.00 (4.00,		7.50 (5.00,		7.00 (4.00,		
	11.00)		12.75)		11.00)		
Range	0.00—26.00		0.00—20.00		0.00—26.00		
Thickness (mm)							0.428
Mean (SD)	19.70 (6.12)		20.48 (6.70)		19.83 (6.22)		
Median (Ǫ1, Ǫ3)	20.00 (16.00,		21.00 (16.25,		20.00 (16.00,		
	24.00)		24.00)		24.00)		
Range	5.00—52.00		8.00—40.00		5.00—52.00		
Brain Atrophy							0.298
No	212 (91.0%)		43 (86.0%)		255 (90.1%)		
Yes	21 (9.0%)		7 (14.0%)		28 (9.9%)		
Nakaguchi grade							0.199
1	49 (21.0%)		9 (18.0%)		58 (20.5%)		
2	74 (31.8%)		16 (32.0%)		90 (31.8%)		
3	69 (29.6%)		21 (42.0%)		90 (31.8%)		
4	41 (17.6%)		4 (8.0%)		45 (15.9%)		
Preoperative extended Nakaguchi classification							0.568
1		20 (8.6%)		4 (8.0%)	24 (8.5%)		
2		18 (7.7%)		3 (6.0%)	21 (7.4%)		
3		11 (4.7%)		2 (4.0%)	13 (4.6%)		
4		73 (31.3%)		16 (32.0%)	89 (31.4%)		
5		34 (14.6%)		9 (18.0%)	43 (15.2%)		
6		36 (15.5%)		12 (24.0%)	48 (17.0%)		
7		41 (17.6%)		4 (8.0%)	45 (15.9%)		
Points of Oslo Grading System according to modifier Nakaguchi classification							0.875
0	98 (42.1%)		20 (40.0%)		118 (41.7%)		
2	135 (57.9%)		30 (60.0%)		165 (58.3%)		
Total points according to Oslo Grading System							***0.037***
0	60 (25.8%)		11 (22.0%)		71 (25.1%)		
1	19 (8.2%)		3 (6.0%)		22 (7.8%)		
2	93 (39.9%)		14 (28.0%)		107 (37.8%)		
3	40 (17.2%)		10 (20.0%)		50 (17.7%)		
4	21 (9.0%)		12 (24.0%)		33 (11.7%)		
Preoperative volume (mL)							***0.002***
Mean (SD)	111.21 (46.80)		133.66 (46.28)		115.18 (47.41)		
Median (Ǫ1, Ǫ3)	114.00 (78.00,		140.50 (93.25,		117.00 (81.00,		
	139.00)		169.75)		146.50)		
Range	7.00—241.00		34.00—212.00		7.00—241.00		
*Treatment Parameters*							
Treatment side							0.876
Left	125 (53.6%)		28 (56.0%)		153 (54.1%)		
Right	108 (46.4%)		22 (44.0%)		130 (45.9%)		
Surgery during anti-platelet anti-coagulant wash-out							***0.01***
No	101 (43.3%)		14 (28.0%)		115 (40.6%)		
Yes	18 (7.7%)		9 (18.0%)		27 (9.5%)		
NP	114 (48.9%)		27 (54.0%)		141 (49.8%)		
Days from anti-platelet anti-coagulant dc to surgery							0.536
N-Miss	10		1		11		
NP	114		27		141		
Mean (SD)	8.98 (14.37)		5.95 (4.95)		8.47 (13.30)		
Median (Ǫ1, Ǫ3)	6.00 (3.00,		4.50 (3.25,		5.00 (3.00,		
	9.00)		7.75)		8.00)		
Range	1.00—90.00		1.00—19.00		1.00—90.00		
Surgery type							0.483
Burr-holes evacuation	205 (88.0%)		42 (84.0%)		247 (87.3%)		
Mini-craniotomy	28 (12.0%)		8 (16.0%)		36 (12.7%)		
Drain							0.483
No	14 (6.0%)		1 (2.0%)		15 (5.3%)		
Yes	219 (94.0%)		49 (98.0%)		268 (94.7%)		
Embolization							** < *0.001***
No	113 (48.5%)		41 (82.0%)		154 (54.4%)		
Yes	120 (51.5%)		9 (18.0%)		129 (45.6%)		
Embolization during anti-platelet anti-coagulant wash-out							0.664
No	43 (18.5%)		3 (6.0%)		46 (16.3%)		
Yes	24 (10.3%)		3 (6.0%)		27 (9.5%)		
NP	166 (71.2%)		44 (88.0%)		210 (74.2%)		
Days from anti-platelet anti-coagulant dc to embolization							0.972
N-Miss	10		0		10		
NP	166		44		210		
Mean (SD)	6.46 (4.95)		6.33 (4.32)		6.44 (4.86)		
Median (Ǫ1, Ǫ3)	5.00 (3.00,		6.00 (3.25,		5.00 (3.00,		
	8.00)		9.50)		8.50)		
Range	1.00—25.00		1.00—12.00		1.00—25.00		
Clinical Parameters at Discharge							
LOS							0.140
Mean (SD)	8.47 (5.82)		8.54 (10.13)		8.48 (6.76)		
Median (Ǫ1, Ǫ3)	7.00 (5.00,		6.00 (4.00,		7.00 (4.00,		
	10.00)		9.75)		10.00)		
Range	1.00—33.00		3.00—55.00		1.00—55.00		
mRS							0.446
0	6 (2.6%)		2 (4.0%)		8 (2.8%)		
1	43 (18.5%)		7 (14.0%)		50 (17.7%)		
2	113 (48.5%)		24 (48.0%)		137 (48.4%)		
3	65 (27.9%)		15 (30.0%)		80 (28.3%)		
4	5 (2.1%)		0 (0.0%)		5 (1.8%)		
5	1 (0.4%)		2 (4.0%)		3 (1.1%)		
Markwalder grading scale							0.699
0	7 (3.0%)		2 (4.0%)		9 (3.2%)		
1	177 (76.0%)		38 (76.0%)		215 (76.0%)		
2	47 (20.2%)		10 (20.0%)		57 (20.1%)		
3	2 (0.9%)		0 (0.0%)		2 (0.7%)		
Complications							0.855
Acute rebleeding	7 (3.0%)		2 (4.0%)		9 (3.2%)		
DVT/PE	10 (4.3%)		2 (4.0%)		12 (4.2%)		
Infection	10 (4.3%)		2 (4.0%)		12 (4.2%)		
No	197 (84.5%)		44 (88.0%)		241 (85.2%)		
Other	8 (3.4%)		0 (0.0%)		8 (2.8%)		
Radiographic Parameters at Discharge							
Postoperative residue cavity volume (mL)							***0.02***
0	172 (73.8%)		29 (58.0%)		201 (71.0%)		
1	61 (26.2%)		21 (42.0%)		82 (29.0%)		
Pneumocephalus > 20% residual volume							0.061
No	124 (53.2%)		19 (38.0%)		143 (50.5%)		
Yes	109 (46.8%)		31 (62.0%)		140 (49.5%)		
*Clinical-Biochemical Parameters at FU*							
mRS							*Not available*
N-Miss	96		0		96		
0	57 (41.6%)		0 (0.0%)		57 (30.5%)		
1	53 (38.7%)		0 (0.0%)		53 (28.3%)		
2	21 (15.3%)		0 (0.0%)		21 (11.2%)		
3	3 (2.2%)		0 (0.0%)		3 (1.6%)		
4	2 (1.5%)		0 (0.0%)		2 (1.1%)		
NP	1 (0.7%)		50 (100.0%)		51 (27.3%)		
Markwalder grading scale							*Not available*
N-Miss	96		0		96		
0	73 (53.3%)		0 (0.0%)		73 (39.0%)		
1	55 (40.1%)		0 (0.0%)		55 (29.4%)		
2	8 (5.8%)		0 (0.0%)		8 (4.3%)		
NP	1 (0.7%)		50 (100.0%)		51 (27.3%)		
Complications other than recurrence							0.344
N-Miss	95		0		95		
No	132 (95.7%)		50 (100.0%)		182 (96.8%)		
Yes	6 (4.3%)		0 (0.0%)		6 (3.2%)		
What?							*Not available*
N-Miss	94		0		94		
DVT	1 (0.7%)		0 (0.0%)		1 (0.5%)		
Empiema	2 (1.4%)		0 (0.0%)		2 (1.1%)		
Seizure	3 (2.2%)		0 (0.0%)		3 (1.6%)		
NP	133 (95.7%)		50 (100.0%)		183 (96.8%)		
Timing re-assumption anti-coagulation therapy							0.526
N-Miss	42		14		56		
1	26 (13.6%)		8 (22.2%)		34 (15.0%)		
2	8 (4.2%)		0 (0.0%)		8 (3.5%)		
5	2 (1.0%)		0 (0.0%)		2 (0.9%)		
6	2 (1.0%)		0 (0.0%)		2 (0.9%)		
NP	153 (80.1%)		28 (77.8%)		181 (79.7%)		
Timing re-assumption anti-platelet therapy							0.377
N-Miss	40		14		54		
1	11 (5.7%)		5 (13.9%)		16 (7.0%)		
2	18 (9.3%)		2 (5.6%)		20 (8.7%)		
3	12 (6.2%)		1 (2.8%)		13 (5.7%)		
4	3 (1.6%)		0 (0.0%)		3 (1.3%)		
6	4 (2.1%)		0 (0.0%)		4 (1.7%)		
NP	145 (75.1%)		28 (77.8%)		173 (75.5%)		
Follow up length (d)							< 0.001
N-Miss	29		0		29		
Mean (SD)	130.06		46.90 (78.25)		113.69		
	(118.80)				(116.65)		
Median (Ǫ1, Ǫ3)	90.50 (57.00,		29.00 (20.25,		76.00 (43.00,		
	146.75)		43.00)		130.00)		
Range	21.00—647.00		6.00—544.00		6.00—647.00		

### Statistical analyses and the machine learning framework

Starting from a dataset comprising 45 clinical, radiological, and laboratory variables across 283 subdural hematomas, a subset of 10 predictors was selected. The nine reported above were identified through statistical significance (p < 0.05) across inferential tests (Kruskal–Wallis, Fisher and Cochran-Armitage) conducted on the full dataset. Based on prior scientific research, one variable (post-operative pneumocephalus of more than 20% of the residual volume) was included literature [24, 25].

These variables were used in two multivariate models: CART and RF.

**Fig. 2:**
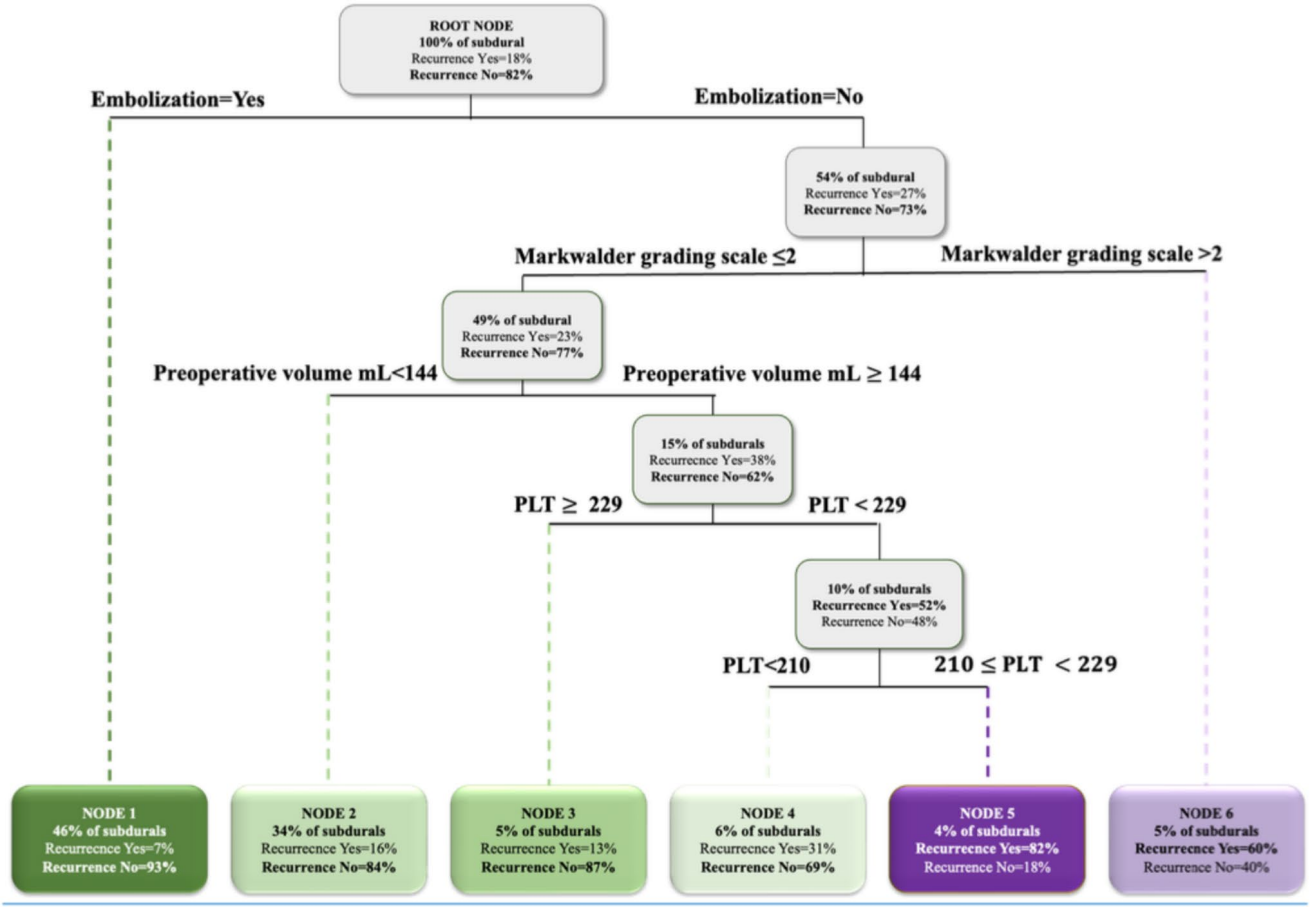
CART model for predicting the recurrence requiring reoperation. The classification tree provides a hierarchical visualization of how chronic subdural hematomas cases were partitioned into increasingly homogeneous subgroups according to recurrence status. The root node aggregates all 283 subdural hematomas included in the study. At each branching point, the algorithm selects the most discriminative covariate along with an optimal cut-off value to split the data into two subsets, thereby enhancing within-node purity. The selected variable and its corresponding rule are displayed above each bifurcation. Father nodes are shown as grey rectangles, indicating the distribution of recurrence versus non-recurrence within each subgroup, with the predominant outcome highlighted in bold. To mitigate the risk of overfitting and preserve model interpretability, tree expansion was constrained through pruning, yielding a structure of optimal complexity. The final level of the tree consists of six terminal nodes (colored rectangles), each encoding a definitive decision rule. Nodes denoting a high probability of recurrence are shaded in purple, while those indicating low risk appear in green. Colored dashed lines connect parent nodes to their child branches, visually tracing the progression from general to increasingly specific clinical profiles. Importantly, the most informative variables appear at the top levels of the tree, underscoring their central role in stratifying recurrence risk

**Fig. 3:**
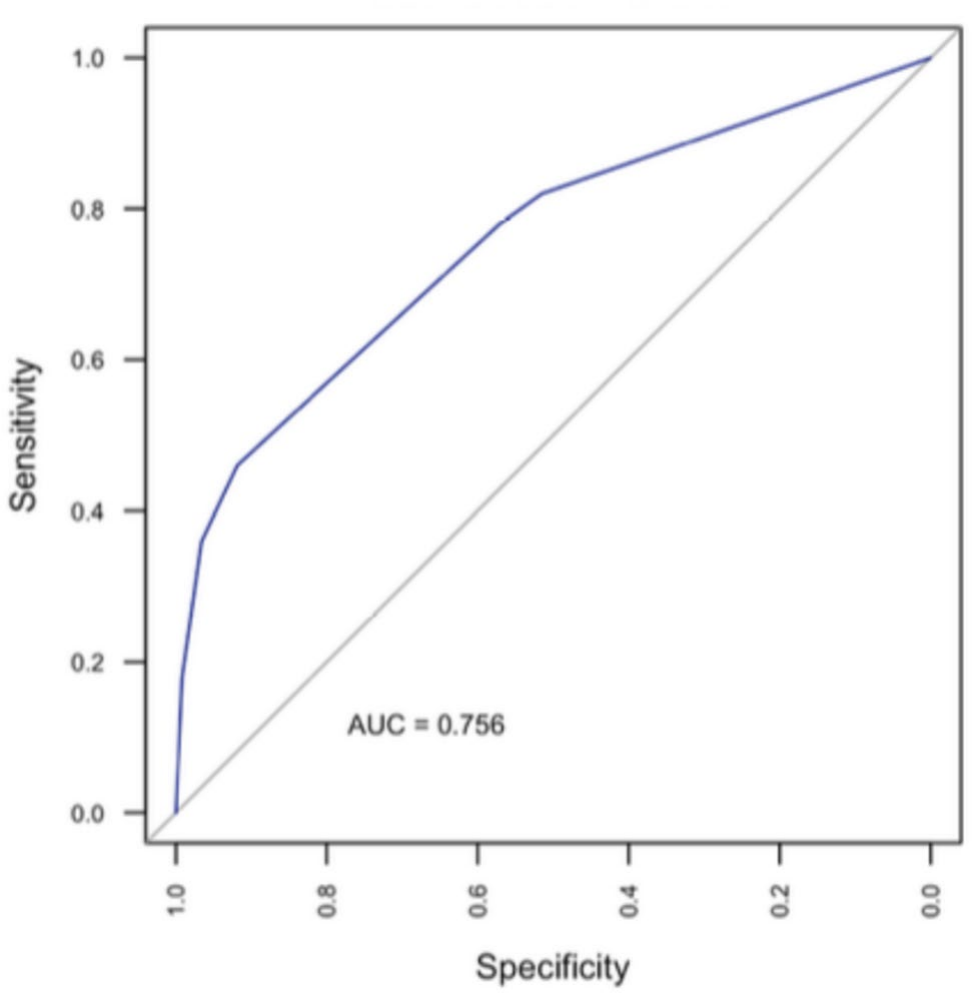
ROC curve for the CART model. The model achieved an AUC of 0.756 and an Accuracy of 0.837, suggesting moderate discriminative performance. CART: Classification and Regression Tree; ROC: Receiver operating characteristic

The CART model stratified subdural hematomas based on preoperative and admission characteristics of the patients, notably Embolization, Markwalder grading scale, V1, and PLT (Fig. 2; see caption for details). The decision tree showed that subdural hematomas undergoing embolization had a very low recurrence rate (7%, Node 1). On the other hand, if non-embolized subdural hematomas exhibited a Markwalder grading scale of ≤ 2 and a V1 of < 144 mL, the recurrence rate was again low (16%, Node 2). In contrast, among non-embolized individuals, if the Markwalder grading scale was greater than 2, the recurrence risk increased to 60% (Node 6). This probability further increased, reaching up to 82% (Node 5), with the Markwalder grading scale ≤ 2, the V1 $\ge$ 144 mL, and 210 × 10⁹/L ≤ PLT < 229 × 10⁹/L. The model achieved an AUC of 0.756 and an Accuracy of 0.837 (Fig. 3).

**Fig. 4:**
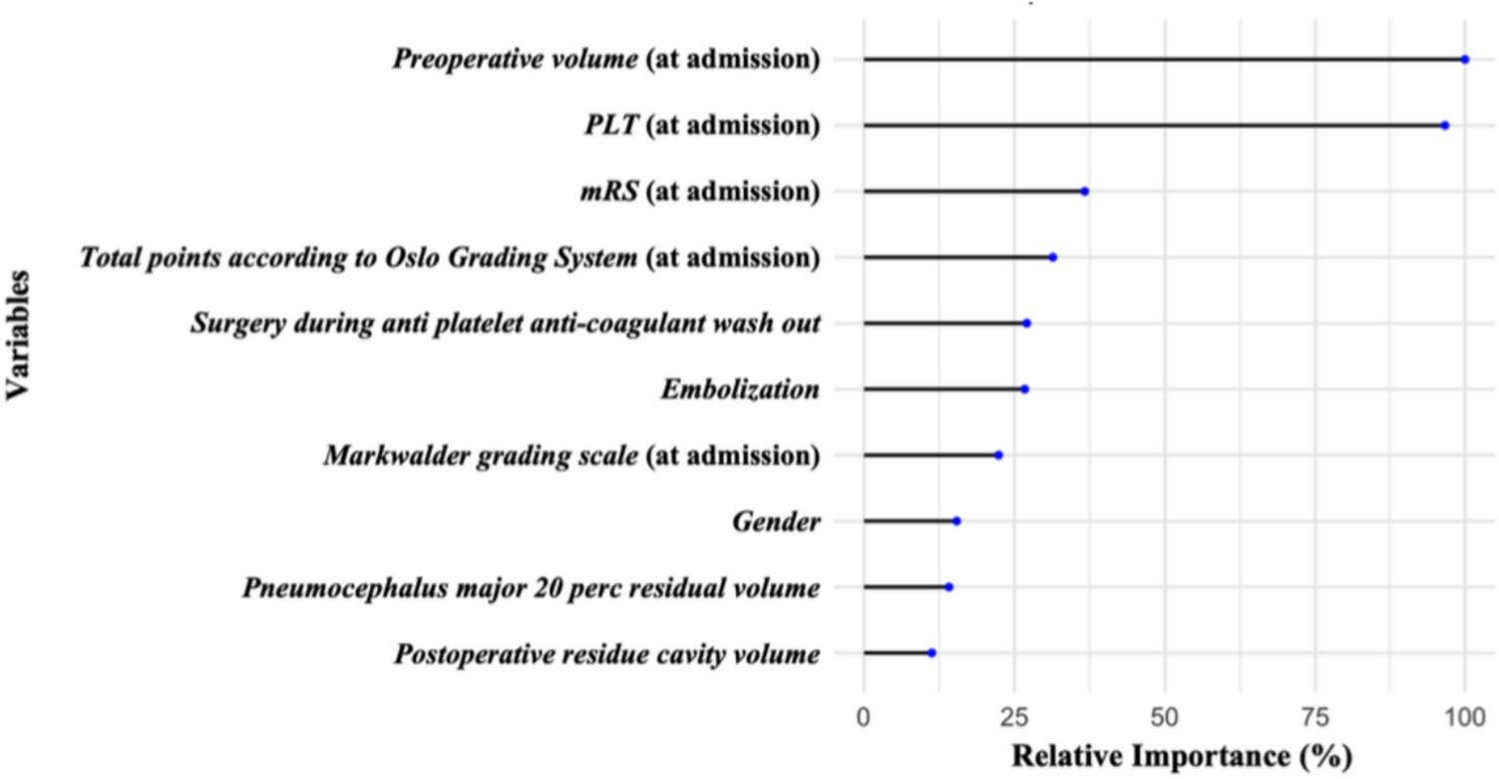
Relative Variable Importance obtained from a RF. The lollipop plot displays the relative importance (in percentage) of ten predictors used in the Random Forest (RF) model, which was trained to classify the risk of recurrence requiring reoperation. Importance scores were computed as the mean decrease in Gini impurity across all decision trees of the Random Forest, normalized with respect to the most influential variable. Each predictor is displayed along the vertical axis, ordered from top to bottom in descending order of relative importance. For each variable, a horizontal black line extends from zero to its importance value, terminating in a blue dot that highlights the specific contribution of the variable to the model’s performance. This visual format enhances readability while maintaining quantitative precision. The three most influential predictors are preoperative volume, PLT and mRS indicating that these covariates are the strongest discriminators for recurrence risk in this cohort. PLT: platelet count; mRS: modified Rankin scale; RF: Random Forest

The RF model, trained on the imputed dataset to handle missing values, provided a ranking of important variables (Fig. 4). The three most influential predictors include V1 (at admission), PLT (at admission), and mRS. These findings are consistent with the structure revealed by CART and highlight the role of radiological, hematological, and neurological parameters in predicting surgical recurrence.

**Fig. 5:**
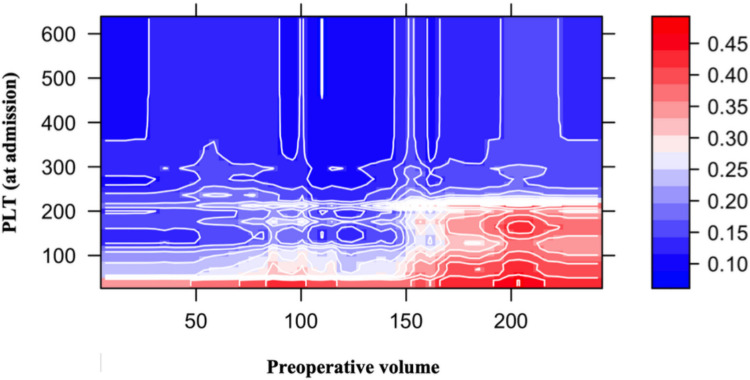
Graphical visualization (2D contour plot) of the PDP on the two most important predictors obtained from a RF. The 2D contour plot displaying the partial dependence of recurrence probability (requiring reoperation) on the two most influential predictors identified by the RF model: Preoperative volume and PLT (at admission). Each point in the plot represents the estimated probability of recurrence as a function of these two variables, while all other covariates are held constant. Warmer colors (shades of red) indicate higher predicted risk, whereas cooler colors (shades of blue) denote lower risk. The contour lines delineate iso-probability regions, allowing for visual interpretation of non- linear interactions between the predictors. The probability of recurrence increases in the lower PLT range (approximately 100 × 109/L—200 × 109/L) when combined with high Preoperative volume (> 150 mL), highlighting a synergistic effect between these two clinical variables. This visualization enhances model interpretability by capturing complex, non-additive relationships typical of ensemble learning methods. PDP: Partial dependence plot; RF: Random Forest; PLT: platelet count

**Fig. 6:**
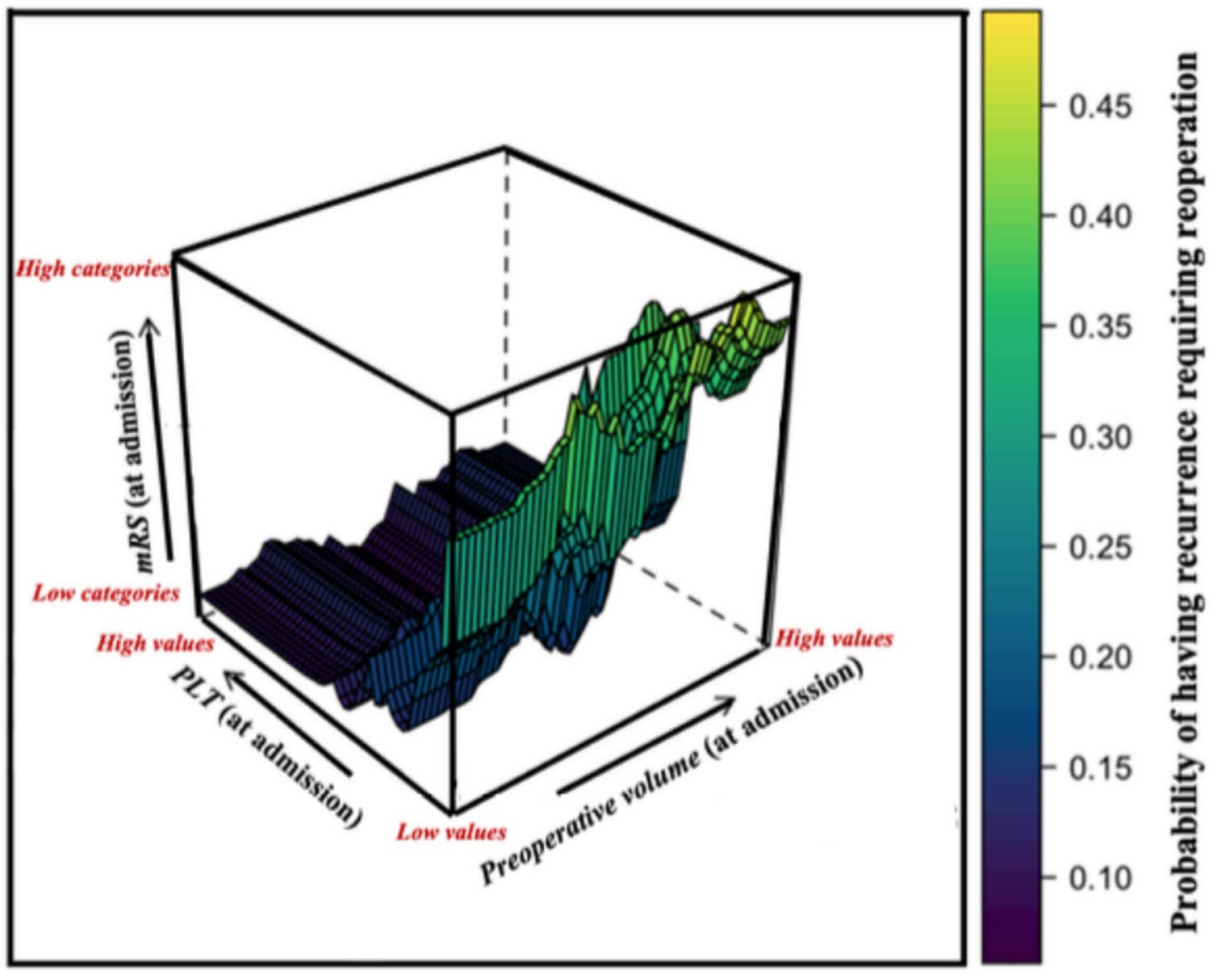
Graphical visualization (3D surface plot) of the PDP on the three most important predictors obtained from a RF. Three-dimensional surface plot derived from partial dependence analysis of the RF model, illustrating the predicted probability of recurrence requiring reoperation as a function of the three most influential predictors: Preoperative volume, PLT (at admission), and mRS (at admission). The surface depicts how the estimated recurrence risk changes across the combined space of these variables, while averaging out the effects of all remaining covariates in the model. Color gradients, from purple (low risk, less than 10%) to yellow (high risk, 40–45%), encode the magnitude of predicted recurrence probability. The plot reveals a clear non-linear interaction between preoperative volume and PLT, with the highest recurrence probabilities observed in cases characterized by large hematoma volumes, intermediate PLT levels, and elevated mRS scores (3 and 4) at baseline. This visualization offers insight into complex multi-dimensional risk patterns not readily apparent through traditional bivariate analysis, supporting the interpretability of ensemble machine learning in clinical outcome modeling. PDP: Partial dependence plot; RF: Random Forest; PLT: platelet count; mRS: modified Rankin Scale

**Fig. 7:**
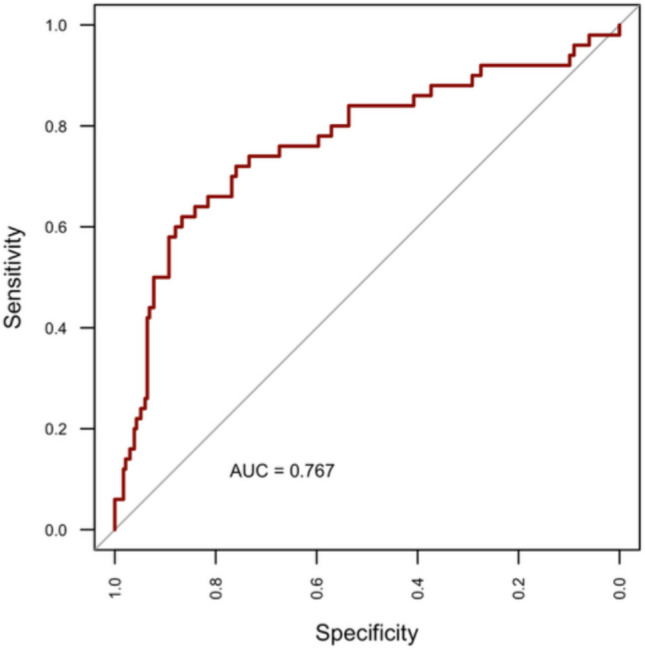
ROC curve for RF model. The ROC curve derived from the RF classifier yielded an AUC of 0.767 and an Accuracy of 0.823, indicating slightly superior predictive capability relative to CART

Finally, to explore interaction effects among these influential features, the PDPs were computed, illustrating the relationships between RF-predicted recurrence probabilities and the top-ranked covariates. The 2D contour plot (Fig. 5) underlines that for high values of V1 (> 150) and low values of PLT (< 100), the predicted probability of recurrence reaches 45%. An interactive version of this plot is available at the following link, allowing users to rotate the image and better visualize the peak in recurrence probability. The 3D surface plot (Fig. 6) adds a variable (mRS): when this third dimension is introduced, it becomes evident that the predicted probability of recurrence markedly increases for higher mRS categories (3 and 4, i.e., stuporous and comatose status, respectively), indicating that greater baseline disability further amplifies recurrence risk. The ROC curve derived from the RF classifier yielded an AUC of 0.767 (Fig. 7) and an Accuracy of 0.823.

## Discussion

Evacuation of cSDH is limited by a significant recurrence rate, ranging from 2.5 to 50% [5, 8, 11, 16, 23, 29], regardless of the surgical technique, which varies from burr hole evacuation to minicraniotomy [9]. Multiple morphological and coagulative risk factors for recurrence have been previously highlighted [10, 13, 15, 22, 24, 29, 33]. The introduction of MMAE in adjuvant therapy with surgical evacuation has significantly reduced its incidence, which has been reported to range from 0 to 14% [5, 8, 11]. However, no studies have comprehensively and hierarchically considered the factors associated with recurrence.

In this study, a CART and RF were used to model the complex relationship between clinical and imaging variables and the risk of subdural hematoma recurrence. The rationale for selecting these supervised machine learning algorithms lies in their ability to flexibly capture non-linear associations and high-order interactions among covariates used in the model, often inadequately addressed by traditional parametric models such as logistic regression (which suffers from non-convergence problems). Indeed, clinical phenomena such as postoperative recurrence are likely influenced by intricate combinations of baseline characteristics rather than isolated linear effects.

CART models offer a particularly appealing strength in their intuitive decision-making process. Through recursive binary partitioning, they construct interpretable decision pathways that group subdural hematomas into risk-based clusters using a hierarchical sequence of clinically intuitive rules. This tree-based representation facilitates the translation of predictive insights into practical clinical decision-making.

Similarly, RF classifiers, despite being ensemble methods with high computational complexity, provide robust predictive performance while mitigating overfitting through aggregation of decorrelated decision trees. In fact, RFs are traditionally considered black-box models. To overcome this limitation, variable importance rankings and PDPs were used, which represent powerful tools for interpreting the results. These visual analytics bridge the gap between algorithmic complexity and clinical usability, enabling domain experts to intuitively grasp how specific variables and their interactions influence recurrence risk.

By integrating such visualization strategies, we can enhance both the transparency and accessibility of advanced machine learning tools, thereby fostering their adoption in multidisciplinary medical contexts where interpretability is crucial.

The most salient finding of the present case series is indeed the hierarchical approach, which showed that embolization has the highest importance in preventing cSDH recurrence, as shown by the CART model (Fig. 2). The same model also documented that a preoperative MGS greater than 3, a V1 less than 144 mL, and a PLT greater than 229 × 10^9^ were protective factors in the non-embolized group. The model exhibits good reliability (AUC = 0.756) with an optimal negative predictive value of 91.7% (Fig. 3).

Nine RrR out of 129 cSDH were observed, with a significant reduction compared to non-embolized patients (p-value < 0.001), which is in line with the most recent literature [5, 8, 11]. However, the prevalence of RrR in the embolized group (7%) is slightly higher than that reported by Davies et al. (4.1%) [5]. This could be explained by a possible selection bias in the latter study, since patients with a MGS of at least 3 were excluded from the analysis. As reported in our study, the MGS differed significantly between the RrR and non-RrR groups (p-value = 0.010) and had a relative importance of slightly less than 25% in the RF, independent of the adjuvant embolization. This is also in line with a previously published CART model, conducted in the pre-embolization era, which showed the MGS as the most relevant factor for unfavourable outcome [25].

Another potential confounding factor is the use of PVA as the embolizing agent. While ethylene vinyl alcohol (EVOH) copolymers are reported to have a higher degree of distal penetration and radiopacity, allowing for more controlled embolization under fluoroscopic guidance [18], a statistical advantage of PVA over EVOH has been reported in terms of last follow-up cSDH thickness in a large case-series by Shehabeldin et al. [27]. In addition, we consider PVA embolization safer in elderly patients because it can be performed under local anaesthesia, being virtually painless when injected. Although ultra-selective MMAE embolization techniques might be accessible, at our center, PVA remains highly valued for its ease-of-use, documented effectiveness, and feasibility under local anesthesia.

Preoperative cSDH volume consistently predicted recurrence across all analyses, and specifically, it was the most impactful factor in the RF. In the non-embolized group, the CART model identified a threshold of 144 mL as the most significant predictor of recurrence in the population with an MGS of less than 2 (Fig. 2). This cut-off is in line with Stanišic et al.'s previous reports [28, 29] (130 and 115 mL). These were derived from a general population that did not consider the patient’s clinical status and may be the reason for the slightly higher value that resulted in our study. Vargas et al. employed a 3-dimensional deep learning automated segmentation pipeline to measure preoperative and postoperative residual volume. The Youden index applied to the AUC identified 140 mL as the critical threshold, which substantially overlaps our finding [31]. While these papers were all conducted in the pre-embolization era, they did not consider patients’ clinical parameters.

In addition to V1, the RF indicated that the PLT was the second factor, with a relative importance of slightly less than 100% (Fig. 4). Observing 3D PDPs (Figs. 5 and 6) and the CART model (Fig. 2), it was underlined that for V1 greater than 144 mL, the PLT showed a critical cut-off at 229 × 10^9^/L for the risk of RrR, independently of embolization. To the best of our knowledge, this is the first paper to highlight the interaction between V1 and PLT, and the critical importance of the latter when preoperative cSDH volume is substantial. The role of PLT has been previously highlighted in the literature: Eagle et al. performed a hierarchical analysis of coagulation factors associated with the risk of reoperation, highlighting PLT as the most important among them [10]. The authors demonstrated a retreatment rate of 12.2% in patients with a PLT between 157 and 313 × 10^9^/L, which is close to the percentage identified in the Node 3 (Fig. 2) (13%) where non-embolized cSDH had a MGS ≤ 2, V1 ≥ 144 mL, and PLT ≥ 229 $\times$ 10^9^/L. This reaffirms the importance of correctly stratifying the risk of reoperation by taking a comprehensive view of clinical, volumetric, and coagulation parameters. Finally, the mean time to reoperation was 46.9 (SD 78.25) days, which is in line with previous works [8, 9, 29], highlighting the importance of close clinico-radiological monitoring if the risk of RrR is significant. In our cohort, the median LOS was 7 days, which appears somewhat longer compared to previous studies [12]. Nonetheless, around 50% of the patients were on ongoing AC and/or AP therapy at the time of hospitalization, requiring a wash-out period before surgery when their neurological status allowed. This significantly extended the LOS.

To summarize, the main strength of this case series is the hierarchical statistical analysis and the comprehensive consideration of multiple and multidisciplinary risk factors for cSDH recurrence. In addition, volume measurements were performed by an experienced neuroradiologist using a contouring technique, which adds robustness to the data presented. Finally, our research applies to daily clinical practice, as embolization is accessible in most centres and should be offered to every patient, PLT can be easily assessed and corrected if necessary, and a multidisciplinary evaluation, which involves neurosurgeons, neuroradiologists and interventional neuroradiologists, would optimize cSDH management.

### Limitations

The primary limitation is the study retrospective nature. The need of collecting complete data for the dataset led to the exclusion of a large amount of patients, especially in the non-MMAE group, leading to a notable attrition. Data regarding restarting AC and/or AG therapies lack in 55 cSDH. Despite being a limitation, numerous earlier studies have shown that quickly resuming AC/AG therapies following cSDH treatment does not elevate the risk of RrR.

Although PVA particles are effective and safe, previous papers have reported a higher, although not statistically significant, percentage of procedural failure and need for reoperation as compared to EVOH [27]. The effect of pneumocephalus on RrR may have been underestimated as data was dichotomised, as in several previous publications [24].

The small sample size and existing extensive studies in the literature precluded an analysis of subgroups within the MMAE-treated population. An analysis of the decision-making algorithm for membranous cSDH was considered; however, due to the small sample size, it was not conducted and is planned for future studies.

## Conclusions

This retrospective single-center series comprehensively and hierarchically evaluated clinical, radiological, coagulation, and treatment factors associated with cSDH recurrence requiring reoperation.

Adjuvant embolization emerged as the strongest and most significant factor associated with recurrence, with a strong protective role. Markwalder grading scale, preoperative cSDH volume, and PLT are strong predictors in non-embolized patients.

The present results support the routine use of MMAE in patients with cSDH and underline that the correction of the PLT in relation to cSDH volume may be beneficial.

## Data Availability

The last author possesses the dataset and can provide it for verification.

## Abbreviations

AC: Anticoagulation
AP: Antiplatelet
aPTT: Activated Partial Thromboplastin Clotting Time
CART: Classification and Regression Tree
cSDH: Chronic subdural hematoma
EVOH: Ethylene vinyl alcohol
LOS: Length of hospital stay
MGS: Markwalder grading scale
MICE: Multivariate imputation by chained equations
MMA: Middle meningeal artery
MMAE: MMA embolization
mRS: Modified Rankin Scale
PDPs: Partial dependence plots
PLT: Platelet count
PVA: Polyvinyl alcohol
RF: Random Forest
ROC: Receiver operating characteristic
RrR: Recurrence requiring reoperation
V1: Preoperative volume
V2: Postoperative residual volume
V3: Last follow-up hematoma volume
